# Therapeutic Strategies for Poststroke Neurological Behavior: From Molecular to Cellular

**DOI:** 10.1155/2018/6215348

**Published:** 2018-10-28

**Authors:** Hailiang Tang, Anatol Manaenko, Qin Hu

**Affiliations:** ^1^Department of Neurosurgery, Huashan Hospital, Fudan University, Shanghai 200040, China; ^2^Friedrich-Alexander-Universität Erlangen-Nürnberg, Erlangen, Germany; ^3^Shanghai Jiao Tong University, Shanghai, China

Ischemic or hemorrhagic stroke usually leads to neurological deficits. Thus, improvement of neurological behavior after stroke is an important task. And neurological behavior is an important indicator of the efficacy of most pharmacological therapies in postischemic/hemorrhagic prognosis. Potential strategies include promoting neuronal regeneration, attenuating neuronal death, mitigating neuroinflammation, and decreasing peripheral immune cell infiltration.

The purpose of this special issue “Therapeutic Strategies for Poststroke Neurological Behavior: From Molecular to Cellular” is to provide an overview of the recent studies for poststroke therapies. The special issue focused on poststroke therapies both in basic and clinical researches, how they improve neurobehavioral function in both animal models and patients, and the underlying mechanisms of pharmacological interventions.

This special issue published 12 selected papers regarding the above specific topics, and the details are summarized below.

Q. Huang et al. did a survey on the relationship of body mass index (BMI) and migraine for Chinese adult people. J. Tian et al. used magnetic resonance imaging (MRI) to evaluate Notch signaling-mediated angiogenesis in ischemic rat. L. Wu et al. found that Biochanin A could reduce inflammatory injury and neuronal apoptosis following subarachnoid hemorrhage (SAH). R. Lan et al. proved that Xiao-Xu-Ming decoction could reduce mitophagy activation and improve mitochondrial function in cerebral ischemia and reperfusion injury. J. Qu et al. found that MST1 suppression could reduce early brain injury after subarachnoid hemorrhage in mice. G. Wang et al. proved that PPAR promoted hematoma clearance in a rat model of intracerebral hemorrhage. Y. Xu et al. reviewed the pathogenesis of necroptosis-dependent signaling pathway in cerebral ischemic disease. H. Yang et al. demonstrated that histone deacetylase inhibitor could alleviate neurological dysfunction after experimental intracerebral hemorrhage in mice. D. Ke et al. proved that hypertriglyceridemia was associated with reduced leukoaraiosis severity in patients with small vessel stroke. L. Wen et al. performed a single-center study for the long-term efficacy and safety of carotid artery stenting among the elderly patients in China. B. Zhu et al. proved that inhibition of GRASP65 phosphorylation protected against cerebral ischemia-reperfusion injury. And moreover, L. Chen et al. studied the effect of carotid artery morphological variations on cognitive function.

We guest editors expect that this special issue will provide readers available data of recent advances in poststroke therapies and may stimulate interest for further research in this area.

